# Supplementary material for: Intensity Modulated Proton Beam Therapy versus Volumetric Modulated Arc Therapy for Patients with Nasopharyngeal Cancer: A Propensity Score-Matched Study
Source: Cancers (Basel). 2021 Jul 16;13(14):3555. doi: 10.3390/cancers13143555 (PMC8307135; doi:10.3390/cancers13143555)
Supplement: Supplementary file 1 [file cancers-13-03555-s001.zip › cancers-1243521-supplementary.pdf]

cancers MDPI

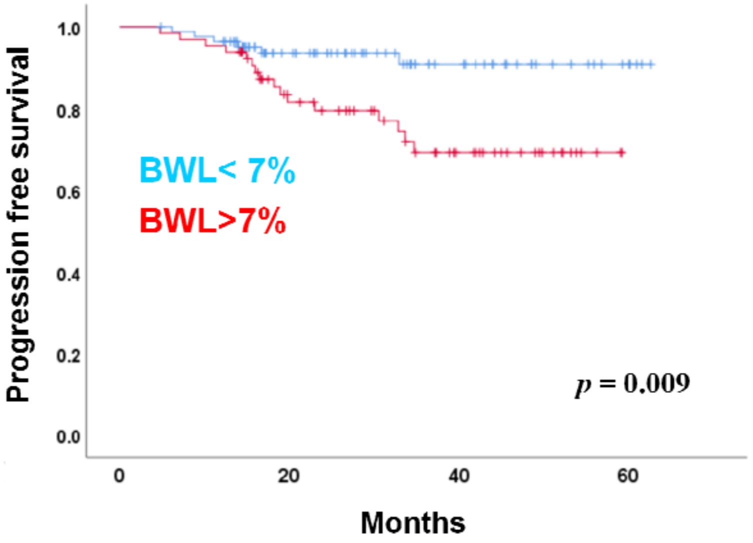

Figure S1. Progression-free survival according to body weight loss during radiotherapy. BWL: Body weight loss

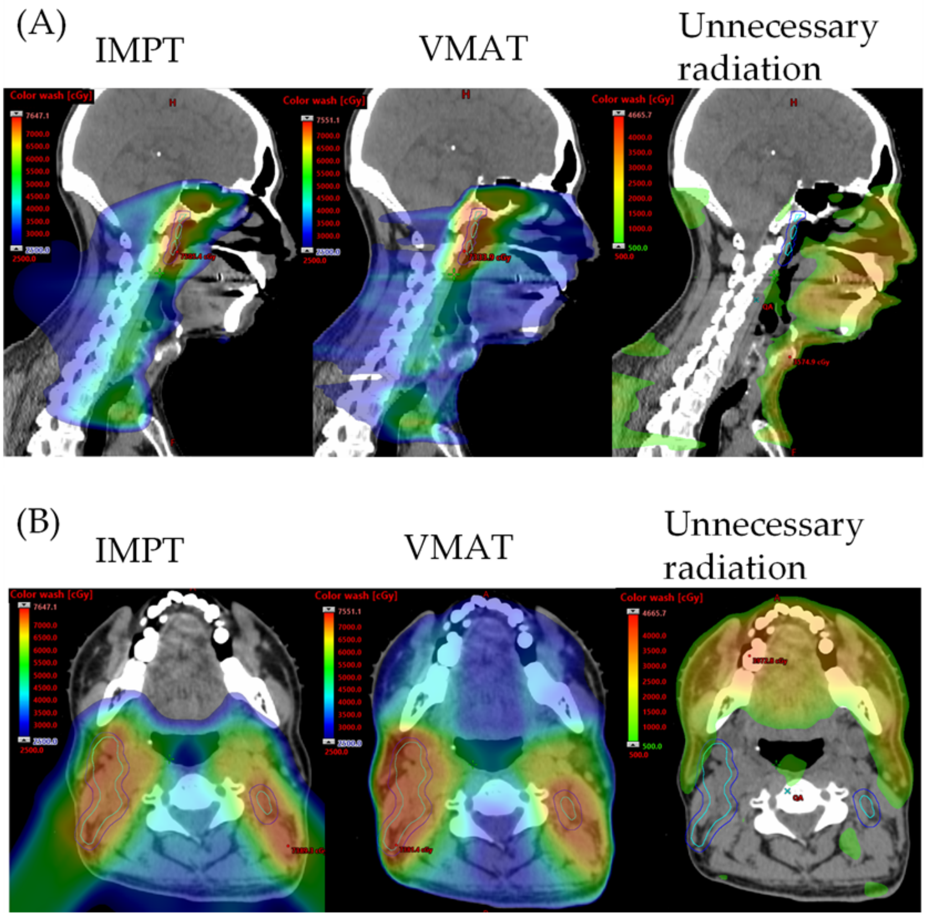

Figure S2. Representative figures regarding IMPT and VMAT treatment planning dose in the same patient with nasopharyngeal cancer. The IMPT reduces unnecessary radiation to the oral cavity. (A) Sagittal view and (B) axial view. Abbreviations: IMPT, intensity-modulated proton therapy; VMAT, volumetric modulated arc therapy

Table S1. The differences in dose coverage and conformity index between the IMPT and VMAT plans.

Target	IMPT(Mean± SD)	VMAT(Mean± SD)	*p*-value
**PTV 6996**			
V100%[%]	91.25±3.52	94.89±3.73	<0.001
CI	1.091±0.130	1.109±0.156	0.449
**PTV5940**			
V59.4 Gy[%]	95.50±2.49	93.95±2.68	0.094
CI	1.311±0.135	1.149±0.141	<0.001
**PTV5412**			
V54.12 Gy[%]	95.06±2.25	91.29±3.84	<0.001
CI	1.246±0.128	1.180±0.127	0.005

IMPT: Intensity-modulated proton therapy, VMAT: Volumetric modulated arc therapy, CI: conformity index.

Table S2. The differences in mean dose to organs at risk between the IMPT and VMAT plans.

Structure	IMPT (IQR)	VMAT (IQR)	*p*-value
Mean oral cavity dose Gy, mean (IQR)	19.8 (6.57–45.01)	38.6 (27.72–58.38)	<0.001
Mean sup. constrictor muscle dose Gy, mean (IQR)	52.9 (16.0–73.78)	61.3 (43.9–76.23)	<0.001
Mean mid. constrictor muscle dose Gy, mean (IQR)	32.0 (7.63–50.6)	42.5 (27.7–66.6)	<0.001
Mean inf. constrictor muscle dose Gy, mean (IQR)	28.6 (6.6–48.8)	34.1 (12.1–56.7)	<0.001

IMPT: Intensity-modulated proton therapy, VMAT: Volumetric modulated arc therapy, IQR: Interquartile range.
